# Dissecting two distinct interneuronal networks in M1 with transcranial magnetic stimulation

**DOI:** 10.1007/s00221-020-05875-y

**Published:** 2020-07-13

**Authors:** Danny Spampinato

**Affiliations:** Department for Clinical and Movement Neurosciences, Institute of Neurology, University College of London, London, UK

**Keywords:** Non-invasive brain stimulation, Transcranial magnetic stimulation, Motor cortex, Learning, Connectivity, Plasticity

## Abstract

Interactions from both inhibitory and excitatory interneurons are necessary components of cortical processing that contribute to the vast amount of motor actions executed by humans daily. As transcranial magnetic stimulation (TMS) over primary motor cortex is capable of activating corticospinal neurons trans-synaptically, studies over the past 30 years have provided how subtle changes in stimulation parameters (i.e., current direction, pulse width, and paired-pulse) can elucidate evidence for two distinct neuronal networks that can be probed with this technique. This article provides a brief review of some fundamental studies demonstrating how these networks have separable excitatory inputs to corticospinal neurons. Furthermore, the findings of recent investigations will be discussed in detail, illustrating how each network’s sensitivity to different brain states (i.e., rest, movement preparation, and motor learning) is dissociable. Understanding the physiological characteristics of each network can help to explain why interindividual responses to TMS exist, while also providing insights into the role of these networks in various human motor behaviors.

## Introduction

Stimulating the brain with transcranial magnetic stimulation (TMS) is a complex process that results in a cascade of descending neuronal activity. This complexity is appreciated when considering that TMS-induced currents activate several axons stemming from various neuronal populations (Rothwell 1997). This includes axons under the coil, as well as others that project to or from other brain regions while also recruiting both inhibitory and excitatory circuitry. Applying a TMS pulse over the primary motor cortex (M1) is capable of producing responses that can be recorded with surface electromyography in contralateral muscle activity, known as a motor-evoked potentials (MEPs). While the physiological underpinnings of the MEP remain poorly understood, this measure has been the dominant readout to quantify corticospinal excitability in behavioral and clinical neuroscience. For instance, MEPs have been used to mark the involvement of M1 in behavioral studies, as well as for understanding the execution and performance of motor actions. As the MEP is the main outcome measure for many studies, understanding the unique capabilities and features of TMS (i.e., stimulation parameters) can help to provide insights to the distinct circuitry recruited with stimulation that contribute towards the MEP.

### Components of the MEP

The MEP is a compound signal that consists of a series of descending corticospinal volleys that summate at the spinal level (Di Lazzaro and Rothwell 2014). This includes the D-wave, which reflects direct activation of pyramidal axons part of the corticospinal tract and is followed by several I-waves (1 ms apart from the D-wave and each other) that reflect indirect depolarization of axons compromised of excitatory and inhibitory neurons (Ziemann and Rothwell 2000). I-waves can have both monosynaptic (early I-waves) and polysynaptic (late I-waves) projections to the output corticospinal neurons (Ziemann and Rothwell 2000). In other words, the MEP is a global readout that reflects the intrinsic excitability of corticospinal cells, including the summation of distinct neural inputs that project to the corticospinal tract and the activity of spinal circuits that contributes to the overall signal.

### Directional TMS recruitment of corticospinal neurones

**Fig. 1:**
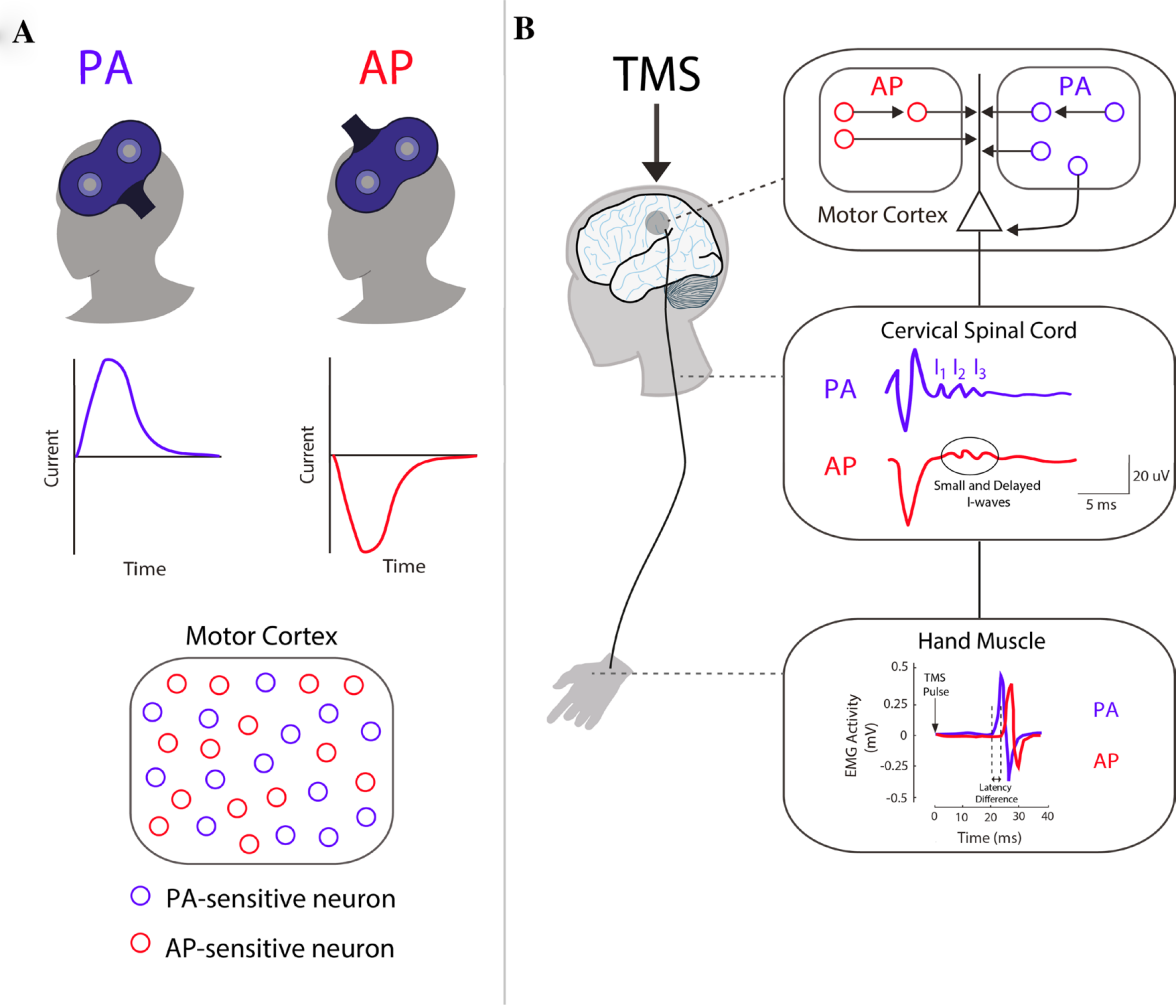
Key differences between PA- and AP-TMS applied to the brain. **a** Diagram depicting the location and position of the TMS coil over the primary motor cortex (M1). Monophasic posterior-to-anterior (PA) and anterior-to-posterior (AP) pulse waveforms are capable of recruiting different subsets of M1 neurons. **b** Stimulation with a figure-of-eight coil over M1 can produce twitches of a desired muscle, which are quantified using electromyography to record the resulting motor-evoked potential (MEP). The descending activity of this complex signal can also be revealed by recording directly from the surface of the spinal cord. This is possible with patients that have electrodes implanted at the high cervical level (~ C2). When a pulse is given with a lateral–medial current (not shown), recordings at the cervical spinal reveal a D-wave, which reflects direct activation of the pyramidal tract. This is followed by a series of indirect waves (I1, I2, and I3) produced from intracortical neurons that are mono- and poly-synaptically connected to pyramidal neurons. Importantly, stimulation with PA currents predominately recruits the I1 wave and is capable of recruiting late I-waves at higher intensities. On the other hand, AP currents produce smaller volleys that are delayed relative to those recruited by PA currents. This is realized when recording the onset of motor-evoked potentials (MEPs) due to brain stimulation. PA currents have been found to consistently evoke faster MEP response when compared to MEPs induced with AP currents (~ 2–3 ms difference). Of note, the responses of AP currents both between and within participants can vary substantially with standard TMS pulse durations

The first evidence that TMS activates a series of distinct activity within M1 was shown by Day et al. (1989) which compared the MEP latency responses evoked by electrical and magnetic stimulation of a targeted hand muscle representation. They found that low stimulation intensities of magnetic stimulation resulted in slightly longer latencies than EMG responses recorded following electrical stimulation. The authors reasoned that magnetic stimulation indirectly activated the corticospinal tract trans-synaptically (i.e., activation through several indirect I-waves), while electrical stimulation only activated direct D-wave axonal responses (Fig. 1). The I-wave threshold responses were found lowest when the current direction was applied in a posterior-to-anterior (PA) direction (Brasil-Neto et al. 1992; Werhahn et al. 1994). Importantly, when the current direction was flipped to an anterior-to-posterior (AP) direction, distinct responses of I-wave components with slightly longer latencies were elicited. Sakai et al. (1997) further expanded upon these results by showing that changes in MEP latency corresponded to changes in the time of recruitment of individual single motor units. With higher stimulation intensities, however, the latency differences evoked by PA and AP currents became obscure. The authors suggested that high intensities recruited the summation of several inputs, and thus, stimulation with low intensities could more effectively activate a particular neuronal network and its specific contribution towards the MEP. While I-waves were initially proposed to reflect the repetitive activity of a neural circuit with excitatory inputs to corticospinal neurons, recent studies (reviewed below) have suggested that stimulation of M1 also activates corticospinal neurons through different subsets of interneuronal circuits.

This knowledge was expanded by Di Lazarro and others (1998), which recorded descending volley activity from epidural electrodes implanted in human participants at the high cervical cord level. They showed that PA currents elicited highly synchronized corticospinal activity that recruited both early and late I-waves, whereas AP currents resulted in less synchronized and slightly delayed corticospinal activity. This result not only explained why differences in latency responses were found, but also demonstrated that current reversal did not simply recruit the reverse the order of descending I-wave activity. Instead, AP stimulation tended to solely recruit late I-waves, albeit with variability across participants. These distinct features of PA and AP stimulation in humans resemble those that have been recorded in animal studies (Patton and Amassian 1954; Kernell and Chien-Ping 1967), including similar interval differences between I-waves (Maier et al. 1997). Even at high intensities where both PA and AP currents can recruit I1, I2, and I3 waves, the latency and variability were slightly longer for AP currents (Di Lazzaro and Rothwell 2014). Together, these studies indicate that AP currents go beyond stimulating a subset of the network activated by PA stimulation as some interneurons unreachable with PA stimulation may be the targets of low-threshold AP stimulation.

These details are important when considering how neurons sensitive to PA and AP currents have shown distinct responses to afferent inputs like short-intracortical inhibition (SICI) and short afferent latency (SAI). SICI, thought to reflect the excitability of intracortical GABAergic circuits (Ziemann et al. 1996), is studied by applying a conditioning pulse a few milliseconds prior to a test pulse over M1 that results in a reduced MEP amplitude. The responses of short-intracortical inhibition (SICI) have been shown to affect mainly the late I-waves that are targeted by AP currents (Hanajima et al. 1998; Sale et al. 2016), which has been recently confirmed in a study showing that threshold tracking with AP currents eliciting a stronger and more robust SICI (Cirillo and Byblow 2016). Similarly, epidural recordings have shown that SAI suppresses late I-waves; however, a greater suppression of MEPs were evoked with PA currents (Ni et al. 2011). The main implication of this study suggests that I-waves activated by PA and AP currents spawned from distinct sources of excitatory inputs. However, if this is the case, future studies limited to using the MEP as a readout will have difficulty in selectively recruiting the distinct inputs to corticospinal neurons. The next section will describe how using the correct combination of conditioned and test pulse parameters can help to differentiate the responses of PA and AP networks to different inputs.

### Selectivity of PA and AP currents

As highlighted, one of the main problems with TMS relates to how stimulation activates various types of neurons (i.e., excitatory vs. inhibitory; interneurons vs. projection neurons), which can explain why MEP responses vary tremendously both within and between individuals. Indeed, while PA currents reliably activate early I-waves, the ability of AP currents to selectivity recruit late I-waves is inconsistent (Hamada et al. 2013). This has an important implication for circuit testing (i.e., SICI and SAI) as individual differences of excitatory I-wave recruitment may also contribute to the interindividual differences seen with conditioning protocols. In other words, differences in interindividual SICI are not necessarily due to differences in the excitability of the GABA system, but rather that the I-waves recruited by the test pulse are less sensitive to the conditioning pulse. This demonstrates how a lack of selectivity can interfere with the interpretation of SICI and other afferent inputs. The question then becomes, are their optimal conditions and TMS parameters that can improve the selectivity of early and late I-wave recruitment?

Recent investigations have made use of the novel controllable TMS (cTMS) device that allows researchers to examine how changes in the pulse waveform (including pulse widths and shapes) can selectively activate particular types of neurons (Delvendahl et al. 2014; D’Ostilio et al. 2016; Hannah and Rothwell 2017; Sommer et al. 2018). Indeed, if PA and AP currents activate different inputs to corticospinal neurons with varying axonal properties, then each network should be sensitive to particular stimulation parameters. D’Ostilio and others showed precisely this as the strength–duration time constants of axons, a measure of how the threshold for stimulation varies with the duration of the stimulus pulse, were found to be different for PA and AP currents. They also found that AP-sensitive inputs were more readily recruited with short pulses (30 us) compared to PA-sensitive inputs with long pulses (120 us), indicating that pulse duration along with current direction determines what excitatory inputs are activated in M1. These results were expanded by Hannah and Rothwell (2017), which showed how applying short-pulse AP currents during voluntary muscle contraction was more selective in producing long latency MEPs (i.e., better recruitment of late I-waves). In this active state, short-pulse AP currents also produced greater SAI responses when compared to both long PA- and AP-pulse widths. This importantly highlights that along with pulse widths, the state of the brain (i.e., rest vs. activity to produce muscle contraction) is critical for improving the selectivity of each network, as muscle activation lowers the threshold (and therefore stimulation intensity) to elicit MEPs. Rather, the high intensities needed for eliciting MEPs in rest conditions recruits a more widespread area of cortex, leading to the recruitment of all I-wave elements (DiLazzaro et al. 2004). Therefore, future study designs will need to consider pulse parameters and muscle activity if the aim is to selectively differentiate the responses of PA and AP networks.

Pulse shape and phase amplitude can also influence response selectivity (Delvendahl et al. 2014; D’Ostilio et al. 2016; Sommer et al. 2018). When considering pulse-phase shapes, it is important to recall that TMS does not deliver a net charge to the cortex, since stimulators recognized as ‘monophasic’ and ‘biphasic’ depend on forward and backward currents. The difference between these pulses is that ‘monophasic’ stimulation creates a brief high amplitude of current followed by a smaller but extended current flow in the opposite direction; ‘biphasic’ stimulation, on the other hand, will have equal lengths and amplitudes of each current flow. As such, biphasic pulses have been shown to recruit a more complex series of inputs than monophasic pulses (Sommer et al. 2018), therefore, a more precise selection of neural populations can be recruited using monophasic pulses. In other words, future studies should refrain from using biphasic stimulation if the goal is to isolate the distinct neuronal networks.

### Can directional TMS provide insights to the induction of cortical plasticity?

The careful consideration of stimulation parameters and circuit selectivity is critical when considering the effects of neuromodulatory protocols such as fixed frequency (e.g., 1 Hz) repetitive TMS (rTMS) and theta burst stimulation (TBS). Depending on the pattern of stimulation, these protocols can induce either excitatory or inhibitory changes in cortical excitability, thought to be linked to mechanisms of synaptic plasticity (Ridding and Rothwell, 2017). However, a considerable amount of variability in the effects of rTMS and TBS have been found across several studies that can partially be explained by interindividual responses to directional TMS.

Hamada and others (2013) demonstrated that individual’s response to TBS strongly correlated to the recruitment of late I-waves with AP currents, whereas individuals lacking this response were found likely not to show either opposite or no response to TBS. This result implies that repetitive protocols appear to engage the cortical circuitry generating late I-waves. However, it is important to note that prior to the development of cTMS, biphasic pulses have predominately been used for rTMS and TBS protocols. As mentioned in the previous section, this would in turn activate both networks sensitive to PA and AP currents, obscuring any role specific role that these networks have towards inducing plasticity. This partially explains why administering TBS over M1 with different current orientations has produced conflicting results (Talelli et al. 2007; Zafar et al. 2008). As only a few recent studies have investigated plasticity protocols with a cTMS device (Goetz et al. 2016; Halawa et al. 2019), future work will need to systematically investigate whether changing both pulse shape (i.e., monophasic pulses) and pulse duration can effectively reduce interindividual responses to modulatory TMS.

The state of brain also influences how PA and AP inputs respond to plasticity protocols. For instance, one study investigating the induction of cortico-cortical plasticity found that pairing parietal cortex stimulation with PA pulses over M1 decreased cortical excitability, whereas pairings with AP pulses increased cortical excitability (Koch et al. 2013). Interestingly, the effects due to parietal pairing with PA pulses was flipped when participants were asked contract a hand muscle, demonstrating that the induction of plasticity within M1 depends on both the stimulation of specific neuronal populations and the activity of the cortex. This idea is supported by a recent study that took advantage of the activity changes that occur within M1 during movement preparation. The authors aimed to induce plasticity by repetitively pairing a TMS pulse 30 ms prior to individuals moving their index finger (i.e., reaction time task). Here, the authors found that administering PA pulses at this specific time induced changes in cortical spinal excitability, whereas no effects were found when the current was reversed (Ibáñez et al. 2019a). This suggests that specific types of movements (i.e. simple motor actions) are more likely to engage neurons that are sensitive to PA stimulation. Future work should consider using movement-related brain stimulation to determine whether different behavioral paradigms (perhaps more cognitively demanding) can specifically target AP-sensitive circuits.

### Are PA and AP networks functionally separated? Evidence from motor learning and movement preparation studies

As the evidence for recruiting separable neuronal networks with directional TMS is clear, one can begin to ask whether specific populations of neurons may contribute to different aspects of motor behaviour. Indeed, it is well known that M1 is engaged in several motor learning processes that are each governed by various physiological mechanisms (Spampinato and Celnik 2017, 2018); therefore, different motor tasks can be used to understand the functional relevance of PA and AP inputs towards motor learning.

Hamada and others (2014) were the first to ask whether priming specifically early (PA-sensitive) or late I-waves (AP-sensitive) excitability could produce dissociable effects on two distinct forms of motor learning. The authors showed that early I-waves facilitated the acceleration of ballistic finger movements (model-free motor learning), whereas late I-waves modulated the learning rate of a cerebellar-dependent visuomotor rotation (model-based learning). While this indicates distinct roles exist for these networks, the method used to probe these circuits was indirect (i.e., plasticity protocols with variable responders). Moreover, the authors claim that PA inputs are independent to cerebellar activity cannot explain how the connectivity between the cerebellum to M1 PA inputs has consistently been shown to respond to model-based learning (Schlerf et al. 2015; Uehara et al. 2017; Spampinato and Celnik 2017, 2018).

**Fig. 2:**
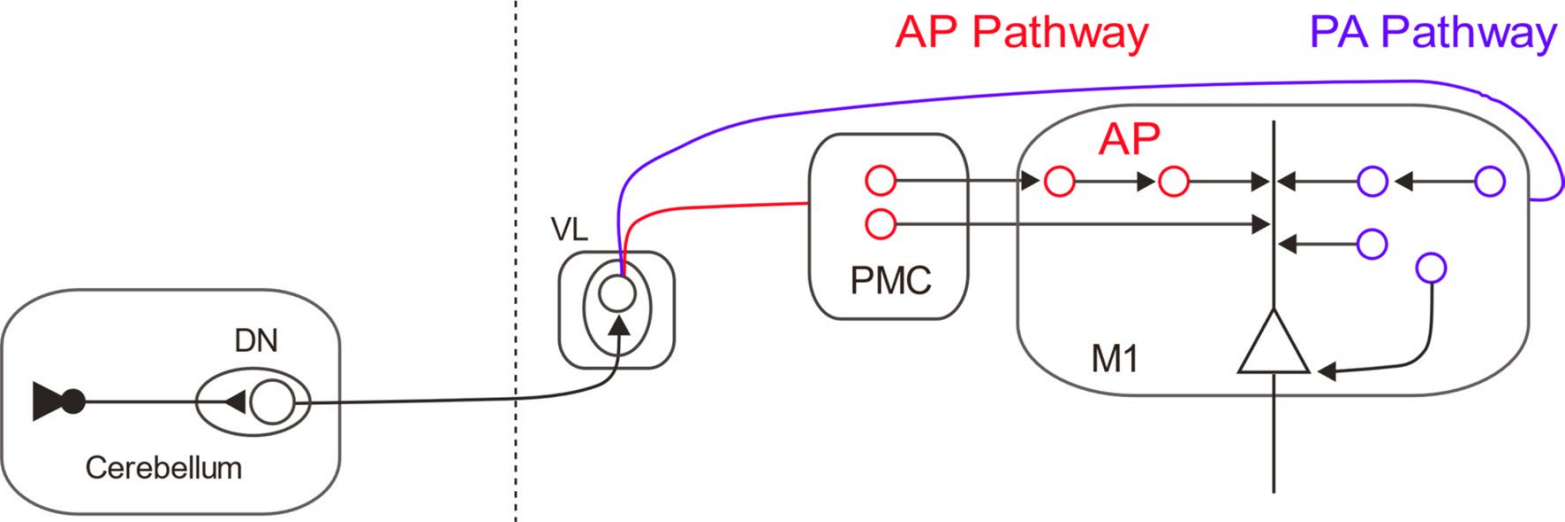
Two distinct cerebellar–cerebral pathways. Schematic depicting two proposed interconnections between the cerebellum and M1. *DN* Dentate nucleus, *VL* ventral lateral section of thalamus, *PMC* premotor cortex. Recent work has shown that separate cerebellar–thalamus pathways interact with both early (e.g., monosynaptic, PA) and late (e.g., polysynaptic, AP) I-waves. It has been suggested that the cerebellar AP pathway may involve projects to the PMC that then are relayed to M1 (Volz et al. 2014; Spampinato et al. 2020). Alternatively, it is possible that back-propagating action potentials within M1 can also play role in the effects described by Spampinato and others (2020), as cerebellar stimulation has been suggested to preferentially affect late I-waves (Iwata and Ugawa 2005; Ugawa et al. 2019)

Recent work has expanded upon this work by showing that two distinct cerebellar-cerebral connections can be disentangled by pairing cerebellar stimulation with directional M1 TMS (Spampinato et al. 2020). Cerebellar connections to PA and AP inputs were found to modulate in distinct ways when individuals were asked to learn two different tasks: (1) a simple motor sequence done with the index finger (involving mainly M1) ; (2) a cognitively-demanding skill task that requires individuals to learn a relationship between a pinch-force production and cursor movement (recruiting premotor areas). Specifically, they showed connections to PA inputs responded to both tasks, whereas AP inputs were only sensitive to the skill learning task that likely engages premotor areas. The authors of this study have proposed that this dissociation of cerebellar–cerebral interactions represents separate cerebellar inputs to the premotor cortex (via AP stimulation) and M1 (PA stimulation; Fig. 2). In support of this, a recent modeling study has shown that AP stimulation may excite premotor regions more so than M1 (Aberra et al. 2019). While this remains speculative, future studies could confirm this idea by applying virtual lesions with TMS over premotor areas and see if cerebellar–cerebral pathways interacting with AP currents are affected. Moreover, this study can serve as a model for future studies utilizing paired-pulse stimulation to understand how different brain regions interact with AP and PA circuits. This will allow for greater understanding of how these distinct interneurons and their associated brain networks may underlie the involvement of specific learning processes (e.g., explicit or implicit learning mechanisms) that are critical for acquiring new behaviors.

A limitation to disentangling the involvement of PA and AP inputs towards motor learning relates to the fact that TMS is used to probe the offline changes in corticospinal excitability. On the other hand, movement preparation to well-known actions presents as an attractive alternative to study these inputs engagement in motor processes for a few reasons: (1) there are considerable fast and dynamic changes in M1 neural activity prior to movement onset (Churchland et al. 2012); (2) the functional role of preparatory activity can be investigated with TMS without the influence of muscle activity. The first TMS experiment applied in this context showed that cortical excitability is reduced during movement preparation (Hasbrouq et al. 1997). While this was initially interpreted as an inhibitory signal that helps to prevent actions from being executed prematurely (Duque and Ivry 2009), the preparatory suppression effect is still a matter of debate (Ibáñez et al. 2019b).

This led Hannah and others to use directional TMS and ask whether the movement suppression extends to both excitatory inputs to corticospinal neurons. The authors reasoned that both inputs should be similarly affected if inhibition for impulse control is responsible for corticospinal suppression. Rather, the authors found that suppression was selective: only MEPs evoked by AP currents were reduced. This suggests that there is a coordinated balance of excitatory inputs (PA-sensitive vs. AP-sensitive) that prevent output neurons from prematurely firing and that AP-sensitive neurons appear to play an important role in suppressing actions. While this work supports the idea that selective suppression of a specific neural networks may be critical for successful movement preparation, future investigations will need to identify the source of this suppression.

In summary, directional TMS allows neuroscientists to investigate how two functionally distinct neuronal circuits contribute to the performance and learning of various motor behaviors. While distinct I-wave components appear to be targeted with different currents, it is important to note that the precise mechanism underlying I-wave generation remains largely misunderstood. Future modeling and computational neurostimulation studies will need to address the nature and origin of I-waves to gain further insights into the properties of the different populations recruited with directional TMS.

### Models of stimulation: what do PA and AP currents recruit?

As it remains unclear as to what areas of the precentral gyrus are most likely activated with TMS, the mechanisms responsible for the delayed differences in I-waves recruited with an AP current compared to PA currents can only be speculated. Possibilities include that (1) an AP stimulus could activate the same axon as those recruited with PA stimulation; (2) a different set of axons (with different conduction times) are activated; (3) sets of cortical neurons arising from distinct cortical regions are recruited. Computational modeling of TMS effects on the cortex has clearly shown that the strength of the induced electric field on the gyral crown is critically affected by current orientation (Tielscher et al. 2011; Opitz et al. 2013), where the strongest field is found when the induced current is perpendicular to the central sulcus. One recent study taking advantage of multi-scale modeling and simulations of the E-fields produced by TMS was able to demonstrate how current direction affected the pattern of neural activation of the precentral gyrus (Aberra et al. 2020). In their model, the authors showed that with AP currents produced an anterior shift of the preferential activation site within the precentral gyrus when compared to PA currents. Their model could account for the longer MEP latencies found in Di Lazzaro et al (2004), while also demonstrating that AP stimulation activated more rostral areas of M1, known to have excitatory inputs from the premotor cortex in both monkeys (Shimazu et al. 2004; Maier et al. 2013) and humans (Groppa et al. 2012). This led the authors to suggest that current direction could activate different cortical circuits, stemming from different brain regions, to generate corticospinal activity. Recent work combining functional magnetic resonance imaging with directional TMS echoed this idea by showing that greater functional connectivity between premotor areas and M1 in individuals which responded best to AP stimulation (Volz et al. 2015). Although this result supports the idea that I-waves generated from AP currents may originate from premotor regions, future studies will need to provide evidence of causality between the interneuronal activity recruited with AP currents and functions dominated by the premotor cortex (e.g., generation of movements). Furthermore, while this review highlights the results of TMS current reversal applied to M1, it remains vastly misunderstood how distinct currents affect non-motor brain regions. The combination of TMS-EEG presents as an attractive approach to investigate how current direction affects local and widespread change in brain neurophysiology, through the recording of TMS-evoked potentials (Rocchi et al. 2018). Future studies will need to explore whether directional TMS on non-motor regions has similar characteristics to those applied over M1.

### Closing remarks

While the evidence for the effect TMS on the neural activity at the single-cell level remains largely unknown, the contributions of John Rothwell have tremendously advanced our ability to uncover two distinct neural networks with TMS. A majority of the studies reviewed in this article have either come directly through his research group or through collaborations with others. The findings from these studies have greatly impacted how current brain stimulation experiments are conducted, which has helped tremendously to uncover the monosynaptic and polysynaptic drives to corticospinal neurons. Moreover, his guidance has helped to lead the many studies which have uncovered the functional roles of these distinct inputs towards motor behaviors and has also given important insights as to why neuromodulatory techniques produce inconsistent effects across individuals. Although John is entering a new phase of his life with his retirement, his impact on the field will forever be felt in the communities of neurophysiology and motor behavioral neuroscience.
